# Prevalencia de colonización de *Staphylococcus aureus* resistente a la meticilina en personal odontológico: revisión sistemática y metaanálisis

**DOI:** 10.7705/biomedica.5794

**Published:** 2026-04-28

**Authors:** Juana Patricia Sánchez-Villamil, Víctor Alfonso Morales-Peña, Juan Felipe Flórez-Joya

**Affiliations:** 1 Departamento de Biología, Facultad de Ciencias, Universidad Antonio Nariño, Bucaramanga, Colombia Universidad Antonio Nariño Departamento de Biología Facultad de Ciencias Universidad Antonio Nariño Bucaramanga Colombia; 2 Facultad de Odontología, Universidad Antonio Nariño, Bucaramanga, Colombia Universidad Antonio Nariño Facultad de Odontología Universidad Antonio Nariño Bucaramanga Colombia

**Keywords:** *Staphylococcus aureus* resistente a meticilina, metaanálisis, odontología, portador sano, prevalencia, Methicillin-resistant *Staphylococcus aureus*, meta-analysis, dentistry, carrier state, prevalence

## Abstract

**Introducción.:**

*Staphylococcus aureus* resistente a la meticilina es un agente patógeno transmitido en hospitales y comunidades. Aunque la presencia en personal médico y de enfermería ha sido ampliamente estudiada, existe poca información sobre su presencia en personal odontológico.

**Objetivo.:**

Estimar la prevalencia de portadores sanos de este agente patógeno en el personal odontológico.

**Materiales y métodos.:**

Se buscaron artículos en PubMed, ScienceDirect, Scopus, BVS y Google Scholar, en inglés, portugués y español. Tres revisores extrajeron los datos de los estudios transversales publicados hasta diciembre del 2024. El riesgo de sesgo se evaluó con la herramienta *Joanna Briggs Institute*. La prevalencia agrupada y los subanálisis se calcularon mediante un modelo de efectos aleatorios con estimación de máxima verosimilitud, utilizando Stata 18.0™. El protocolo fue registrado en PROSPERO (CRD42024563164).

**Resultados.:**

Se incluyeron 17 artículos en la revisión sistemática y 6 en el metaanálisis. La prevalencia general de presencia nasal de *S. aureus* resistente a la meticilina en personal odontológico fue del 2,28 % (IC_95%_: 0,78-6,4), de un total de 1256 participantes. En odontólogos, la prevalencia fue del 3,63 % (IC_95%_: 0,98-10,57); en asistentes dentales, del 1,45 % (IC_95%_: 0,11-11); y en estudiantes en prácticas clínicas, del 2,0 % (IC_95%:_ 0,36-8,0). No se observaron diferencias significativas entre los grupos (p = 0,87).

**Conclusiones.:**

Los estudios de prevalencia de portadores sanos de *S. aureus* resistente a la meticilina en personal odontológico son escasos y sin representatividad poblacional. A pesar de una baja prevalencia, la vigilancia rigurosa de todos los proveedores de atención médica permite el monitoreo de su transmisión epidemiológica y los factores de riesgo predisponentes.

## Abstract (en)

**Introduction.:**

Methicillin-resistant *Staphylococcus aureus* is a pathogen transmitted both in hospital and community settings. Although carriage among medical and nursing personnel has been widely studied, information regarding its presence among dental healthcare workers is limited.

**Objective.:**

To estimate the prevalence of healthy methicillin-resistant *S. aureus* carriers among dental personnel.

**Materials and methods.:**

We searched for articles in PubMed, ScienceDirect, Scopus, BVS, and Google Scholar in English, Portuguese, and Spanish. Three reviewers selected and extracted data from cross-sectional studies published up to December 2024. We assessed risk of bias using the Joanna Briggs Institute tool. We calculated pooled prevalence and conducted subgroup analyses using a random-effects model with maximum likelihood estimation in Stata 18.0™. The protocol was registered in PROSPERO (CRD42024563164).

**Results.:**

We included 17 articles in the systematic review and six in the meta-analysis. The overall prevalence of nasal methicillin-resistant *S. aureus* carriage among dental personnel was 2.28% (95% CI: 0.78-6.4) in a combined sample of 1256 participants. Among dentists, prevalence was 3.63% (95% CI: 0.98-10.57); among dental assistants, 1.45% (95% CI: 0.11-11), and among dental students in clinical training, 2.0% (95% CI: 0.36-8.0). Differences between groups were not statistically significant (p = 0.87).

**Conclusions.:**

Evidence on methicillin-resistant *S. aureus* carriage among dental personnel is scarce and lacks population representativeness. Although prevalence appears low, continued surveillance among all dental Healthcare providers is recommended to monitor epidemiological transmission and associated risk factors.

Al igual que en cualquier otra profesión en salud, la práctica odontológica pone a los profesionales de esta área en riesgo de problemas relacionados con la salud ocupacional. Los dos problemas más prevalentes son los trastornos musculoesqueléticos (espalda, cuello y hombros) y las lesiones percutáneas ^1^^,^^2^. La exposición a fluidos corporales como la saliva y la sangre los pone en riesgo de adquirir agentes infecciosos, no solo por contacto directo, sino también por la presencia de partículas invisibles suspendidas en el aire, denominadas bioaerosoles.

Las piezas de alta velocidad en odontología generan bioaerosoles de diferentes tamaños desde nanómetros hasta 100 micrómetros, que pueden contener partículas bacterianas, virales y fúngicas ^3^. Los bioaerosoles llevan los microorganismos de la cavidad oral al ambiente. En estudios previos se evidenció el incremento de los bioaerosoles en los consultorios justo durante la visita de los pacientes ^4^, así como también en los ductos de los instrumentos dentales de irrigación, donde estas bacterias forman biopelículas ^5^.

Un importante desafío, tanto para el personal sanitario como para la salud pública, es la circulación de bacterias resistentes a varios tipos de antibióticos ^6^. La resistencia bacteriana a los antimicrobianos es una de las principales causas de muerte a nivel mundial, estimadas en 1,14 millones para el 2021 ^7^.

El principal agente patógeno Gram positivo involucrado es *Staphylococcus aureus* resistente a la meticilina ^7^^,^^8^. Esta bacteria es la principal causa de infección nosocomial en Latinoamérica ^9^ y genera particular interés en los países europeos, donde se ha observado un incremento de su prevalencia en infecciones, tanto en entornos comunitarios como sanitarios ^10^.

En los consultorios odontológicos, *S. aureus* es una bacteria de importancia considerable puesto que se encuentra frecuentemente en la cavidad oral ^11^^,^^12^ y, por lo tanto, se ha hallado involucrada de forma oportunista en infecciones orales ^13^ y complicaciones posteriores a la colocación de implantes ^14^.

En un estudio reciente realizado en Alemania sobre el perfil microbiológico en infecciones odontogénicas, se aisló esta bacteria en el 5,8 % de los casos ^15^.

Los profesionales de la salud, incluyendo los odontólogos, pueden adquirir *S. aureus* resistente a la meticilina y otras bacterias superresistentes, por medio de los bioaerosoles y materiales o superficies contaminadas, y pueden portarlo de forma asintomática. La presencia asintomática de bacterias resistentes a antibióticos por parte del personal de atención en salud es un problema de salud pública porque actúan como reservorios, propagando rápidamente la bacteria durante la atención de pacientes y entre la comunidad ^16^.

Actualmente, no contamos con un dato preciso del perfil epidemiológico de *S. aureus* resistente a la meticilina entre los profesionales de odontología, que permita determinar si tienen o no un papel importante como reservorios dentro de la comunidad, o si el ambiente odontológico tiene relevancia en la transmisión específica de esta bacteria.

Por tanto, se realizaron una revisión sistemática y un metaanálisis con el objetivo de integrar, tanto la información existente sobre la prevalencia de portadores sanos de esta bacteria entre el personal de odontología, como las características metodológicas de los estudios.

## Materiales y métodos

La presente revisión sistemática se llevó a cabo siguiendo la guía *Preferred Reporting Items for Systematic Reviews and Meta-Analyses* (PRISMA) para el reporte de revisiones y metaanálisis ^17^. La pregunta de investigación que se pretendía responder fue: «¿Cuál es la prevalencia de portadores sanos de *Staphylococcus aureus* resistente a la meticilina en personal de odontología?».

### Métodos de búsqueda para identificar los estudios

La búsqueda se realizó en las siguientes bases de datos: Pubmed, ScienceDirect, Scopus, Biblioteca Virtual de Salud (SciELO, LILACS) *y* Google Scholar. Se utilizaron los siguientes términos MeSH/DeCS: *Staphylococcus aureus*, *methicillin-resistant Staphylococcus aureus*, *health personnel*, *students*, *dental*, *dentists*, *students*, *health occupations*, *carrier state/status*, *nasal carriage y colonization*.

### Criterios de inclusión y exclusión de los estudios

Los criterios de inclusión fueron: estudios originales, de corte transversal, publicados hasta diciembre del 2024, en inglés, español o portugués. Los estudios debían reportar la prevalencia de colonización por *S. aureus* resistente a la meticilina en muestras obtenidas en nariz, nasofaringe, boca o manos en profesionales de odontología, estudiantes en práctica clínica, higienistas dentales y auxiliares o asistentes en odontología.

Se excluyeron las publicaciones de tipo póster y los comentarios o notas en editoriales, al igual que aquellos estudios cuya unidad de análisis fueron los aislamientos individuales y no los sujetos examinados.

### Extracción y análisis de datos

Los tres autores realizaron de forma independiente la búsqueda y selección de los artículos en cada una de las bases de datos tras la revisión de títulos y resúmenes. Luego, se revisó el texto completo de los estudios seleccionados. Los autores evaluaron el riesgo de sesgos y la extracción de datos y, ante cualquier desacuerdo, se llevaron a cabo consensos.

Se recopiló en un archivo de Excel la siguiente información para cada artículo: país donde se realizó la investigación, año de publicación de los resultados, tamaño de la muestra, tipo de muestra obtenida, grupo poblacional (estudiante, graduado, posgrado o asistente/auxiliar/técnico), estimaciones de prevalencia (totales o estratificadas por tipo de muestra o técnica de identificación), técnicas de identificación y evaluación de resistencia a otros antibióticos, incluyendo la vancomicina.

### Evaluación del riesgo de sesgo

La calidad metodológica de los estudios fue evaluada usando la *Joanna Briggs Institute Clinical Appraisal Checklist*^18^. Se tuvo en cuenta la valoración de nueve criterios, que tenían dos posibilidades de calificación: 0, si no contaba con la suficiente calidad, y 1 en caso de que sí la tuviera. Con esta herramienta, se evaluó si había sesgos en el diseño, el desarrollo o el análisis de los estudios. También, se hizo una evaluación subjetiva de la calidad, en la que se calificaba como alta (> 8 puntos), moderada (6-8 puntos) o baja (0-5 puntos).

Respecto a la evaluación de riesgo de sesgo, Cochrane recomienda la evaluación subjetiva del riesgo para el caso de estudios observacionales en los que se examina la prevalencia, ya que no existe un consenso actual ^19^. Teniendo en cuenta lo anterior, se revisaron los criterios dispuestos en la lista de chequeo de forma independiente.

### Análisis estadístico

Para el metaanálisis se utilizó el *software* Stata 18,0™ (Stata Corp LP, College Station, Texas). A partir de los estudios incluidos, se extrajo el reporte de las estimaciones de prevalencia como dato cuantitativo y se realizó la clasificación por el tipo de ocupación (odontólogo, auxiliar o asistente, estudiante en prácticas) y el tipo de muestra obtenida.

Se utilizó una estadística descriptiva para los datos de prevalencia. Para las muestras de hisopado nasal, el cálculo se realizó agrupando los valores por tipo de ocupación, con el respectivo valor del intervalo de confianza del 95 %.

Para el valor de la prevalencia agrupada, se eligió el modelo de efectos aleatorios de DerSimonian-Laird y la transformación doble arco seno de Freeman-Tukey con el objetivo de darle una distribución normal a la varianza de las proporciones.

La heterogeneidad entre los estudios se evaluó mediante el estadístico I^2^ y el diagrama de Galbraith. Con un valor de I^2^ de 40 a 60 %, la heterogeneidad se consideró moderada y, con uno mayor de 60 %, se consideró alta.

## Resultados

### Selección de los estudios

Se encontraron 818 artículos en las bases de datos, aplicando las estrategias de búsqueda diseñadas, y 2 artículos más a partir de la bibliografía de artículos primarios. Una vez seleccionados por título y resumen, el número de artículos se redujo a 46 y, finalmente, a 20 al eliminar los duplicados.

Una vez revisado el texto completo, se excluyeron tres artículos: uno por no reportarse *S. aureus* resistente a la meticilina, ya que su objetivo era evaluar la colonización por *S. aureusy* la producción de enterotoxina ^20^, y otros dos porque fue imposible obtener los datos ^21^^,^^22^. Entre estos últimos se encontraba el estudio de Krishnan Thantry ^22^, ya que los autores no discriminaron entre estudiantes y profesionales. En este estudio, se reportó ausencia de *S. aureus* resistente a la meticilina en 40 muestras de hisopado nasal.

**Figura 1:**
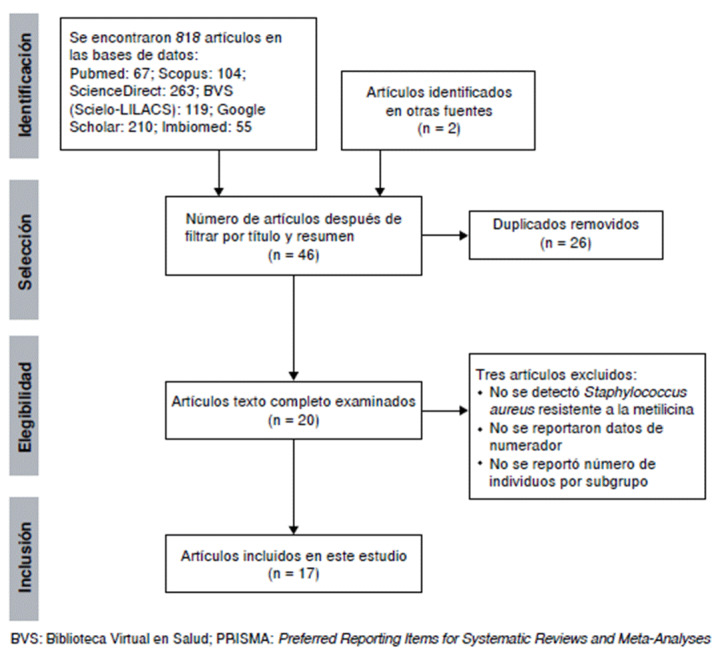
Diagrama de flujo PRISMA para la selección de artículos

Finalmente, se incluyeron 17 estudios, de los cuales se extrajeron y analizaron los datos. El flujo de estudios de esta revisión y metanálisis se presenta en la figura 1.

Los estudios incluidos fueron publicados del 2011 en adelante. De acuerdo con la ubicación geográfica, de los 17 estudios incluidos: 7 (41,2 %) se llevaron a cabo en países de Asia, con 834 sujetos muestreados; 4 (23,5 %) en Europa, con 1155 sujetos; y 6 (35,3 %) en América, en 644 sujetos. Esto da un total de 2633 sujetos de los servicios de odontología incluidos en estudios de prevalencia de la bacteria resistente, de los cuales 918 (34,9 %) eran odontólogos profesionales.

La metodología de aislamiento e identificación de *S. aureus* resistente a la meticilina varió entre los diferentes estudios. En 5 (31 %) estudios, se utilizó el agar cromogénico para la selección diferencial; en 10 estudios (62,5 %), se implementó la prueba de sensibilidad a oxacilina-cefoxitina; y en otros 10, se utilizaron pruebas moleculares para detectar genes de resistencia a las penicilinas.

Estas pruebas no se aplicaron de forma exclusiva. En el 60 % de los estudios, aproximadamente, se utilizaron dos técnicas para detectar y confirmar la presencia de dicha bacteria. En el 31 % de los estudios, también se evaluó la resistencia a la vancomicina mediante técnicas tradicionales o moleculares, con solo un estudio con reporte positivo del 2 % en muestras de hisopado nasal. En el cuadro 1 se presentan las características principales de los estudios incluidos.

**Cuadro 1:** Características de los estudios incluidos en la revisión sistemática

	Autor, año	País	N	Tipo muestra	Tipo de personal	**Técnica de identificación de *S. aureus***	Técnica detección MRSA
1	Roberts MC *et al.*, 2011	Estados Unidos (Washington)	61	Hisopado nasal	Estudiantes	No realizado	Recuperación caldo Bacto Staph + 75 mg/L polimixina. Agar selectivo y cromogénico selección de MRSA BioRad SCCmec typing
2	Martínez-Ruiz *et al.*, 2014	México	100	Hisopado nasal y faríngeo	Estudiantes	Cultivo recuperación en TSA 37 oC por 16 horas - Agar manitol-salado 37 oC por 24 horas	Oxacilina MIC >4 mg/ml y Gen *mecA* positivo
3	Petti *et al*., 2015	Italia	90	Hisopado nasal, palmar y oral	Estudiantes	Toma de muestras en solución salina 4 oC 1 hora - 1 ml agar manitol- salado 37 oC por 48 horas, subcultivo en caldo TSA	Difusión por disco oxacilina y PCR gen *mecA*
4	Baek *et al.*, 2016	Corea (Seúl)	77	Hisopado nasal	Estudiantes	No realizado	Agar ChromID MRSA, PCR *mecA y* SCCmec
5	Kumar y Muralidharan, 2016	India (Chennai)	50	Hisopado nasal	Estudiantes y odontólogos en hospital dental	Morfología de colonias en agar sangre, prueba de coagulasa	Cefoxitina
6	Amado Centenero *et al.*, 2016	Brasil	31 17	Hisopado nasal y saliva	Odontólogos en UCI salado Auxiliares dentales en UCI	Agar manitol	Agar cromógeno con suplemento de cefoxitina y oxacilina y detección de genes *mecA*
7	Hema *et al*., 2017	India	200	Hisopado nasal	Estudiantes de postgrado	No realizado	Agar cromógeno HiMedia suplemento con cefoxitina, 37 oC por 48 horas Confirmación por test de coagulasa y test de difusión de cefoxitina en agar Müeller-Hinton
8	Yoo et al., 2018	Corea (Seúl)	96 25 18	Hisopado nasal	Odontólogos Higienistas Técnicos dentales	No realizado	Agar cromogénico ChromID MRSA bio- Mérieux, difusión por disco oxacilina. Confirmación *mecA* y SCCmec tipificación
9	Pineda Higuita *et al.*, 2020	Colombia	62	Hisopado nasal	Estudiantes de IX y X semestres	No realizado	Cefoxitina y oxacilina
10	Senok *et al.*, 2020	Emiratos Árabes	96 50	Hisopado nasal	Odontólogos Estudiantes	No realizado	Micromatrices de ADN StaphyType Agar cromógeno
11	Cavaco-Silva *et al*., 2021	Portugal	464	Hisopado nasal	Estudiantes	Agar manitol salado, agar cromogénico MRSA y test de coagulasa	Agar MH difusión poor disco de oxacilina 6 mg/ml, 4 % NaCl y cefoxitina
12	Khairalla *et al*., 2021	Egipto	60	Hisopado palmar	Odontólogos	2 ml BHI 37°C x 18-24 horas 100 ul Agar manitol salado, catalasa, coagulasa	Difusión por disco oxacilina (1 μg) y cefoxitina (30 μg). spa y SCCmec tipificación por PCR
13	Nandita Subba Rao et al., 2020	India	59	Hisopado nasal	Odontólogos	Tinción de Gram. Agar BHI, pruebas catalasa y coagulasa	No descrito
14	Salmanov *et al*., 2020	Ucrania	155	Hisopado nasal	Odontólogos generales y especialistas	Automatizado (Vitek-2; bioMerieux)	Cefoxitina 30 μg, genes *mecA* y *femA*
15	Lerche *et al*., 2021	Alemania	297 149 38	Hisopado nasal	Auxiliares Odontólogos Recepcionistas Técnicos en odontología	No realizado	Spa typing
16	Goes *et al*., 2021	Brasil	6	Hisopado nasal	Asistentes dentales y odontólogos	Fenotípica identificación por tinción de Gram, pruebas de catalasa y coagulasa. Genotípica identificación gen Sa442	PCR mecA y SCCmec oxacilina y cefoxitina
17	García-Herrera *et al*., 2024	Colombia	298 96 26 37	Hisopado nasal	Odontólogos general y especialistas Auxiliares Higienistas orales Estudiantes	Tinción de Gram, hemólisis, pruebas catalasa y coagulasa. Agar manitol- salado	Cefoxitina 30 μg y gen *mecA*

MRSA: Staphilocuccus aureus resistente a la meticilina

En menos del 50 % de los estudios, se hacía una descripción detallada de las características sociodemográficas de los sujetos incluidos, de los datos específicos del tipo de especialidad y del servicio prestado, lo que limita el análisis por subgrupos o, incluso, su validez externa o aplicabilidad a otros sujetos en otros contextos de atención en salud.

Se observó heterogeneidad en las características del personal muestreado, el tipo de muestra y las técnicas microbiológicas de identificación.

Respecto al sesgo de información, se encontró que en dos estudios no se aplicaron los métodos de las CLSI establecidas para identificar *S. aureus y S. aureus* resistente a la metilicina, y evaluar su sensibilidad a los antimicrobianos.

### Prevalencia de Staphylococcus aureus resistente a la metilicina

En los resultados de la revisión sistemática se encontró que los estimados de prevalencia en portadores nasales oscilaron entre el 0 % en todos los subgrupos y el 28,5 % en estudiantes, el 35 % en auxiliares o técnicos y el 23 % en odontólogos. Esto no incluye el estudio de Goes *et al*. ^23^, en el cual se encontraron 4 positivos en un grupo de 3 odontólogos y 3 auxiliares odontológicos.

En general, el número de estudiantes tamizados (1146) fue mayor que el de odontólogos, técnicos y auxiliares examinados, el cual no supera el 78 % del primero.

En su mayoría, el número de datos de estudiantes provinieron de estudios realizados en América. Los datos de auxiliares y profesionales de odontología provinieron de estudios realizados en Alemania ^24^, Corea ^25^ y los Emiratos Árabes ^26^.

Las prevalencias más altas del agente patógeno se observaron en estudiantes, con un valor máximo del 35,7 %, seguidos por grupos de odontólogos y auxiliares, con valores máximos del 29 y el 17 %, en estudios llevados a cabo en países de Latinoamérica y en los Estados Unidos.

En los estudios de Petti *et al*.^27^^),^ Khairalla *et al*.^28^^)^ y Amado *et al*.^29^^),^ también se detectó la bacteria en manos, boca y saliva, de forma simultánea al muestreo de hisopado nasal. Los valores de prevalencia en dichas localizaciones anatómicas fueron menores que los observados en las fosas nasales.

En Italia, Petti *et al*. estudiaron un grupo de estudiantes de odontología; si bien no se identificó *S. aureus* resistente a la metilicina, si se encontró *S. aureus* con una prevalencia ligeramente menor en manos [3,3 % (0,3 % - 6,4 %)] y aproximadamente similar en boca [7,8 % (2,2 % - 13,3 %)], en relación con lo reportado en fosas nasales [6,7 % (1,5 % -11,8 %)].

Respecto a la prevalencia de la bacteria resistente a la meticilina en odontólogos de Egipto ^28^ y en auxiliares dentales de unidades de cuidados intensivos en Brasil ^29^, se observaron valores 4,6 y 6 puntos porcentuales por debajo del valor registrado en las fosas nasales, y en manos y saliva, respectivamente

### Valoración de la calidad metodológica

Para eliminar el riesgo de subjetividad individual durante la valoración de la calidad metodológica del conjunto de estudios, primero se hizo una calibración para validar los 9 criterios evaluados y se determinó el acuerdo interevaluador, siendo este del 82,4 %.

En el cuadro 2 se presenta el resumen del riesgo de sesgo de los 17 estudios incluidos. Se encontró que 7 de los 17 artículos eran de buena calidad, con al menos 6 de 9 puntos totales para los criterios de calidad. De los estudios con buen reporte de calidad, se excluyó el estudio de Goes *et al*. ^23^, debido a que solamente se reportaron tres profesionales de atención primaria en odontología seleccionados para tamizaje de *S. aureus* resistente a la metilicina.

**Cuadro 2:** Resumen de la evaluación del riesgo de sesgo utilizando la herramienta JBI para los estudios incluidos

Autor	P1	P2	P3	P4	P5	P6	P7	P8	P9	Puntaje total
Roberts MC, 2011	1	0	0	0	1	1	0	0	0	3
Martinez-Ruiz, 2014	0	0	0	0	0	1	1	1	0	3
Petti S, 2015	1	Población total	0	1	1	1	0	0	1	6
Amado WL, 2016	1	0	0	1	1	1	1	0	0	5
Baek YS, 2016	1	Población total	1	1	1	1	0	1	0	6
Kumar & Muralidharan, 2016	1	0	0	0	0	1	0	0	0	2
Hema, 2017	1	0	0	0	1	1	0	0	0	3
Khairalla, 2017	1	1	1	0	1	1	1	1	0	7
Yoo YJ, 2018	1	0	0	0	1	1	0	0	0	3
Senok, 2019	1	Población total	0	1	0	0	0	0	0	3
Pineda, 2020	1	Población total	0	1	0	1	0	0	0	4
Nandita Subba Rao, 2020	1	0	0	1	1	0	0	1	1	5
Salmanov, 2020	1	1	1	1	1	1	1	0	1	8
Goes *et al*., 2021	1	Población total	0	1	1	1	1	1	0	7
Lerche, 2021	1	1	1	1	0	1	1	1	1	8
Cavaco-Silva *et al*., 2021	1	0	0	0	0	1	0	0	0	2
Garcia-Herrera *et al*., 2024	1	1	1	1	1	1	1	1	1	9

P1-P9 = criterios/preguntas del 1 al 9 de la herramienta JBI. JBI:

*Joanna Briggs Institute*

El mayor inconveniente se observó durante la selección de individuos, ya que eran estudios realizados en profesionales de instituciones universitarias u hospitales, sin previa aleatorización de sujetos. Por lo tanto, los resultados obtenidos son poco precisos y no generalizables a toda la población. Tampoco se registró el reporte de falta de respuesta y no solían reportarse los intervalos de confianza.

En el análisis de gráfico de embudo, se observó asimetría sin uniformidad en su distribución, lo cual sugiere la presencia de sesgo de publicación o de fenómeno de efectos de estudios pequeños ^30^. Dichos hallazgos nos confirmaron que los estudios son efectivamente pequeños y tienen poca calidad metodológica. Esto es consecuencia de que se presenten resultados de estudios con escasos individuos de grupos sectarios en instituciones prestadoras de servicios odontológicos.

## Metaanálisis

De 7 artículos que cumplieron con los criterios para inclusión en el metaanálisis, 6 se utilizaron para extraer los datos y calcular los estimados o valores. Solo se utilizaron los datos con reportes de hisopado nasal. Se excluyeron los resultados de Goes *et al*. ^23^, debido a que su tamaño de muestra fue de tan solo tres individuos. Entre el personal odontológico (n = 1256 sujetos), la prevalencia combinada de portadores nasales sanos de *S. aureus* resistente a la meticilina fue de 2,28 % (IC_95_%: 0,78 - 6,4 %).

El análisis por subgrupos arrojó las siguientes prevalencias:

- en odontólogos: 3,63 % (IC_95_%: 0,98-10,57 %, n = 633);
- en personal asistente: 1,45 % (IC_95_%: 0,11-11 %, n = 419) y
- en estudiantes: 2,03 % (IC_95_%: 0,36-8 %, n = 204).

**Figura 2:**
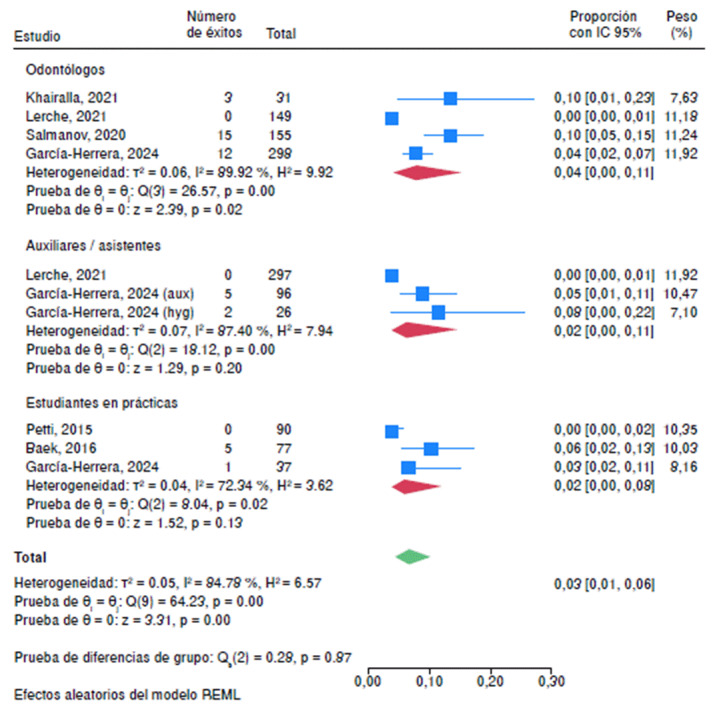
Diagrama de bosque para calcular la prevalencia por subgrupos

Además, el *x* ^2^ no mostró diferencias significativas entre los valores (p =0,87) (figura 2).

El índice de heterogeneidad para cada subgrupo evidenció una considerable variabilidad entre los estudios con I^2^ superiores al 60 %. El análisis mediante el diagrama de Galbraith mostró que cuatro estudios que cayeron fuera de la banda de confianza fueron los que contribuyeron a esa heterogeneidad. Por tanto, los valores agrupados obtenidos no representan un estimado preciso.

## Discusión

En esta revisión, se identificó y se sintetizó la evidencia respecto a la prevalencia de *S. aureus* resistente a la meticilina entre el personal de salud dental, incluyendo odontólogos, estudiantes y asistentes. La revisión sistemática reveló que los estudios enfocados en profesionales de odontología son escasos. La mayoría de estos presentan sesgos de selección debido a la ausencia de una población claramente definida y a la falta de un cálculo del tamaño muestral. Además, gran parte de los datos reportados no reflejan la colonización a nivel poblacional, sino que se limitan a profesionales de una única institución o centro hospitalario, predominantemente clínicas universitarias.

En relación con revisiones previas en personal odontológico, esta es, hasta donde se tiene conocimiento, la primera revisión sistemática y el primer metaanálisis centrados en este grupo profesional. El metaanálisis evidenció una prevalencia promedio de colonización nasal del 2,28 %, cifra comparable con la reportada en estudios comunitarios y distinta a la observada en otros profesionales de salud que laboran en entornos intrahospitalarios. Los resultados no muestran diferencias significativas en la prevalencia de portadores nasales de *S. aureus* resistente a la meticilina según la ocupación del personal odontológico.

En comparación, en revisiones sistemáticas de otras áreas de la salud, se ha reportado una prevalencia global de colonización nasal del 9,23 % en el personal médico y de enfermería en los países asiáticos ^31^. En los Estados Unidos y en los países europeos, las estimaciones oscilan alrededor del 5,4 % (IC_95_%: 4,61 %-6,39 %), con un riesgo de colonización 2,5 veces mayor para enfermeras que para cualquier otro profesional y tasas de prevalencia menores en los países europeos ^32^.

En cierta medida, las diferencias son esperables teniendo en cuenta que el personal odontológico labora en condiciones de atención primaria, donde el riesgo de adquirir una bacteria resistente es menor, pues no son habituales la atención ni la concentración de pacientes con sistemas inmunológicos comprometidos y con exposición prolongada a antibióticos, como sí ocurre en los servicios de hospitalización o en las unidades de cuidado intensivo.

En las publicaciones se afirma constantemente que la principal fuente de contaminación en la clínica odontológica proviene de superficies y equipos instrumentales contaminados, y que la concentración de microorganismos en el ambiente aumenta durante los procedimientos odontológicos ^33^.

Por otra parte, la transmisión de *S. aureus* y *S. aureus* resistente a la meticilina por inhalación de aerosoles generados al toser o al estornudar, es menos frecuente, y los trabajadores de la salud rara vez son fuente de transmisión para los pacientes ^34^. En este contexto, la transmisión aérea mediante aerosoles en la clínica dental es un factor relevante; sin embargo, el uso adecuado de los equipos de protección personal, y la observancia rigurosa de los protocolos de manejo y desinfección del instrumental, constituyen factores fundamentales para la protección del personal. En estudios posteriores a la pandemia del coronavirus del SARS-CoV-2, se demostró que el riesgo de contagio para todo el personal de salud es comparable y menor en el lugar de trabajo que fuera de él ^35^^,^^36^, lo cual está relacionado con el cumplimiento de las normas para el control de infecciones ^37^.

En el análisis de datos, se revisó si había disponibilidad de información concerniente a la especialidad del profesional muestreado, su tiempo de ejercicio profesional y sus factores de riesgo, así como el reporte de antecedentes quirúrgicos y cumplimiento del uso de elementos de protección personal. Sin embargo, la gran mayoría de los estudios tan solo reportan el dato de portador, sin ninguna información adicional particular sobre las características de la población en estudio.

Una razón para esta falta de caracterización puede ser el hecho de que la población esté restringida a un único centro odontológico, hospitalario o institución universitaria, lo que limita el análisis por grupos de diversas variables debido al tamaño de la muestra.

Respecto a las limitaciones de esta revisión, es importante tener en cuenta que no se buscó literatura gris, lo que puede haber dejado sin incluir algunas investigaciones. Además, se observaron varios métodos y protocolos microbiológicos con los que se identificaron *S. aureus y S. aureus* resistente a la metilicina, lo que puede cuestionar la comparabilidad y coherencia de los valores reportados de prevalencia del portador nasal.

Cabe aclarar que ninguno de los métodos es inválido, aunque sí presentan un valor de sensibilidad variable para identificar el fenotipo de resistencia a la meticilina en *Staphylococcus*^38^. Además, los métodos deben ajustarse a los estandarizados por el CLSI, respecto a las técnicas de dilución en caldo o agar, o la técnica de difusión con discos ^39^. También, se debe considerar que tanto el carácter de portador como la expresión fenotípica de resistencia a la meticilina (oxacilina o cefoxitina) bajo diferentes condiciones de cultivo, son transitorios.

Es importante destacar que, exceptuando los estudios de Lerche *et al.*^24^, García-Herrera *et al*. y Goes *et al*. ^23^, ninguno se hizo considerando una población de profesionales en odontología que fuera representativa de una región o país en relación con variables relevantes como edad, sexo, tiempo de desempeño, tipo de ocupación e instituciones donde se labora (García-Herrera YA, Martínez DC, Jiménez VM, García BC, Arias-Mendoza MJ, Cuéllar JO, et al. Prevalencia de portadores sanos de SARM en personal de odontología: primer reporte en Colombia. Memorias, IV Encuentro Internacional de Ciencias de la Salud: la ciencia transforma, Bucaramanga. Revista Salud UIS; 2024. p. 15.),

Teniendo esto en cuenta, se podría desestimar la utilidad del valor agrupado calculado en estos metaanálisis, al no representar el riesgo poblacional de ser portador de *S. aureus* resistente a la meticilina siendo prestador de servicios odontológicos. En vez de eso, dicho valor representa el riesgo de este personal en centros universitarios e instituciones hospitalarias.

Así pues, se genera la necesidad de que en estos estudios de prevalencia se consideren criterios de validez externa como los propuestos en la herramienta de evaluación de riesgo de sesgos elaborada por Hoy *et al.*^40^, en la que se identifica la representatividad, la selección aleatoria y la ejecución de un censo inicial de la población blanco.

Se concluye que existe un número limitado de estudios sobre la prevalencia de portadores nasales de *S. aureus* resistente a la meticilina en el personal de odontología, caracterizados por tamaños muestrales reducidos y enfoques monocéntricos, lo que limita la representatividad de la situación a nivel poblacional en los servicios odontológicos.

El metaanálisis evidencia una prevalencia baja de colonización por *S. aureus* resistente a la meticilina en el personal de servicios odontológicos, en comparación con otros profesionales de la salud. El riesgo de identificar un portador asintomático de dicha bacteria en las fosas nasales del personal odontológico es inferior al 10 %.

Desde la perspectiva de la salud pública, estos resultados indican un riesgo ocupacional bajo en los servicios odontológicos para la propagación de agentes patógenos resistentes. Esto, debido a que el riesgo depende, tanto de la prevalencia de estos agentes patógenos en la población atendida por el personal de odontología, como del uso adecuado de los equipos de protección personal, que durante la práctica clínica son una defensa fundamental para prevenir la adquisición y la transmisión de *S. aureus* resistente a la meticilina por parte del personal de salud.

Se recomienda realizar estudios poblacionales amplios en este grupo de profesionales, que incluyan la recopilación de datos sobre factores de riesgo *y* análisis de secuenciación genómica, incluso de otros agentes infecciosos como los virus de las hepatitis B y C, y del virus de la inmunodeficiencia humana, con el fin de obtener una perspectiva epidemiológica integral. En consecuencia, esta revisión sistemática aporta implicaciones relevantes, tanto para la práctica clínica como para futuras investigaciones en el área.

## References

[1] Moodley R, Naidoo S, Wyk J (2018). The prevalence of occupational health-related problems in dentistry: A review of the literature. J Occup Health.

[2] Reddy V, Bennadi D, Satish G, Kura U (2015). Occupational hazards among dentists: A descriptive study. J Oral Hyg Health.

[3] Kim K-H, Kabir E, Jahan SA (2018). Airborne bioaerosols and their impact on human health. J Environ Sci (China).

[4] Kobza J, Pastuszka JS, Bragoszewska E (2018). Do exposures to aerosols pose a risk to dental professionals?. Occup Med (Chic Ill).

[5] Barbot V, Robert A, Rodier M-H, Imbert C (2012). Update on infectious risks associated with dental unit waterlines. FEMS Immunol Med Microbiol.

[6] Zumla A, Hui DSC (2019). Emerging and reemerging infectious diseases: Global overview. Infect Dis Clin North Am.

[7] Naghavi M, Vollset SE, Ikuta KS, Swetschinski LR, Gray AP, Wool EE (2024). Global burden of bacterial antimicrobial resistance 1990-2021: A systematic analysis with forecasts to 2050. Lancet.

[8] Murray CJ, Ikuta KS, Sharara F, Swetschinski L, Robles Aguilar G, Gray A (2022). Global burden of bacterial antimicrobial resistance in 2019: A systematic analysis. Lancet.

[9] Guzmán-Blanco M, Mejía C, Isturiz R, Álvarez C, Bavestrello L, Gotuzzo E (2009). Epidemiology of meticillin-resistant Staphylococcus aureus (MRSA) in Latin America. Int J Antimicrob Agents.

[10] Di Ruscio F, Guzzetta G, Bjernholt JV, Leegaard TM, Moen AEF, Merler S (2019). Quantifying the transmission dynamics of MRSA in the community and healthcare settings in a low- prevalence country. Proc Natl Acad Sci USA.

[11] Cataldo Russomando K, Jacquett Toledo N, Fariña N, Pereira A, Rodríguez F, Guillen RM (2017). Portación de Staphylococcus aureus multiresistentes a antimicrobianos en cavidad bucal de niños que concurren para un tratamiento en una clínica odontológica, Paraguay. Pediatría (Asunción).

[12] McCormack MG, Smith AJ, Akram AN, Jackson M, Robertson D, Edwards G (2015). Staphylococcus aureus and the oral cavity: An overlooked source of carriage and infection?. Am J Infect Control.

[13] Al-Akwa AAY, Zabara AQMQ, Al-Shamahy HA, Al-labani MA, Al-Ghaffari KM, Al-Mortada AM (2020). Prevalence of Staphylococcus aureus in dental infections and the occurrence of MRSA in isolates. Universal Journal of Pharmaceutical Research.

[14] Rokadiya S, Malden NJ (2008). An implant periapical lesion leading to acute osteomyelitis with isolation of Staphylococcus aureus. Br Dent J.

[15] Meinen A, Reuss A, Willrich N, Feig M, Noll I, Eckmanns T (2021). Antimicrobial resistance and the spectrum of pathogens in dental and oral-maxillofacial infections in hospitals and dental practices in Germany. Front Microbiol.

[16] Cimolai N (2008). The role of healthcare personnel in the maintenance and spread of methicillin- resistant Staphylococcus aureus. J Infect Public Health.

[17] Page MJ, McKenzie JE, Bossuyt PM, Boutron I, Hoffmann TC, Mulrow CD (2021). Declaración PRISMA 2020: una guía actualizada para la publicación de revisiones sistemáticas. Rev Esp Cardiol.

[18] Joanna Briggs Institute (2016). Critical appraisal checklist for analytical cross sectional studies.

[19] Higgins JPT, Thomas J, Chandler J, Cumpston M, Li T, Page MJ (2019). Cochrane handbook for systematic reviews of interventions. Cochrane Database Syst Rev.

[20] Pereira ML, do Carmo LS, Souki M, dos Santos E, Carvalho MA, Bergdoll MS (1999). Staphylococci from dental personnel. Braz Dent J.

[21] Reddy Y (2010). Prevalence of methicillin-resistant Staphylococcus aureus (MRSA) nasal colonization among dental professionals.

[22] Krishnan Thantry A, Ping Sern N, RamNavas S, Ramachandran S, Zin Tan M, Viswanathan N (2016). Surveillance study on nasal and hand carriage of methicillin-resistant Staphylococcus aureus (MRSA) among dental and medical staff and students of a medical university. Oral Health Dent Manag.

[23] Goes ICRS, Romero LC, Turra AJ, Gotardi MA, Rodrigues TFSO, Santos LO (2021). Prevalence of nasal carriers of methicillin-resistant Staphylococcus aureus in primary health care units in Brazil. Rev Inst Med Trop Sao Paulo.

[24] Lerche N, Holtfreter S, Walther B, Semmler T, Al'Sholui F, Dancer SJ (2021). Staphylococcus aureus nasal colonization among dental health care workers in Northern Germany (StaphDent study). Int J Med Microbiol.

[25] Baek YS, Baek S-H, Yoo Y-J (2016). Higher nasal carriage rate of methicillin-resistant Staphylococcus aureus among dental students who have clinical experience. J Am Dent Assoc.

[26] Senok A, Nassar R, Kaklamanos EG, Belhoul K, Abu Fanas S, Nassar M (2020). Molecular characterization of Staphylococcus aureus isolates associated with nasal colonization and environmental contamination in academic dental clinics. Microb Drug Resist.

[27] Petti S, Kakisina N, Volgenant CMC, Messano GA, Barbato E, Passariello C (2015). Low methicillin-resistant Staphylococcus aureus carriage rate among Italian dental students. Am J Infect Control.

[28] Khairalla AS, Wasfi R, Ashour HM (2017). Carriage frequency, phenotypic, and genotypic characteristics of methicillin-resistant Staphylococcus aureus isolated from dental healthcare personnel, patients, and environment. Sci Rep.

[29] Centenero WLA (2016). Ocorrencia de Staphylococcus aureus em saliva e cavidade nasal em abientes hospitalar e odontológico.

[30] Hong C, Salanti G, Morton SC, Riley RD, Chu H, Kimmel SE (2020). Testing small study effects in multivariate meta-analysis. Biometrics.

[31] Giri S, Ghimire A, Mishra A, Acharya K, Kuikel S, Tiwari A (2023). Prevalence of methicillin- resistant Staphylococcus aureus carriage among healthcare workers in South Asia in nonoutbreak settings: A systematic review and meta-analysis. Am J Infect Control.

[32] Dulon M, Peters C, Schablon A, Nienhaus A (2014). MRSA carriage among healthcare workers in non-outbreak settings in Europe and the United States: A systematic review. BMC Infect Dis.

[33] Motta RHL, Ramacciato JC, Groppo FC, Pacheco A de BND, de Mattos-Filho TR (2005). Environmental contamination before, during, and after dental treatment. Am J Dent.

[34] Price JR, Cole K, Bexley A, Kostiou V, Eyre DW, Golubchik T (2017). Transmission of Staphylococcus aureus between health-care workers, the environment, and patients in an intensive care unit: A longitudinal cohort study based on whole-genome sequencing. Lancet Infect Dis.

[35] Natapov L, Schwartz D, Herman HD, Markovich DD, Yellon D, Jarallah M (2021). Risk of SARS-CoV-2 transmission following exposure during dental treatment - A national cohort study. J Dent.

[36] Chea N, Brown CJ, Eure T, Ramírez RA, Blazek G, Penna AR (2022). Risk factors for SARS- CoV-2 infection among US healthcare personnel, May-December 2020. Emerg Infect Dis.

[37] Al-Zoughool M, Al-Shehri Z (2018). Injury and infection in dental clinics: Risk factors and prevention. Toxicol Ind Health.

[38] Brown DFJ, Edwards DI, Hawkey PM, Morrison D, Ridgway GL, Towner KJ (2005). Guidelines for the laboratory diagnosis and susceptibility testing of methicillin-resistant Staphylococcus aureus (MRSA). J Antimicrob Chemother.

[39] Kiehlbauch JA, Hannett GE, Salfinger M, Archinal W, Monserrat C, Carlyn C (2000). Use of the National Committee for Clinical Laboratory Standards guidelines for disk diffusion susceptibility testing in New York state laboratories. J Clin Microbiol.

[40] Hoy D, Brooks P, Woolf A, Blyth F, March L, Bain C (2012). Assessing risk of bias in prevalence studies: Modification of an existing tool and evidence of interrater agreement. J Clin Epidemiol.

